# Fist case of laboratory confirmed BCG infection following vaccination in children in Albania

**DOI:** 10.1186/1753-6561-5-S6-P250

**Published:** 2011-06-29

**Authors:** S Tafaj, G Kasmi

**Affiliations:** 1 National TB Reference Laboratory, Spitali Universitar "Shefqet Ndroqi", Tirana, Albania; 2 Department of Microbiology, University Hospital Center "Mother Teresa", Tirana, Albania

## Introduction / objectives

The attenuated bacilli Calmette-Guerin (BCG) vaccine is administered in Albania to all newborns at the first day of life to prevent tuberculosis. Although complications are rare after BCG vaccination and the outcome is usually favourable, serious BCG infections can occur. The risks associated with BCG vaccination include local complications, extraregional localized disease, and disseminated BCG disease.

## Methods

We report a case of M. bovis BCG infection in an 11-month old immunocompetent girl. The child was referred to the National TB Reference Laboratory by the pediatrician to perform smear examination and culture for mycobacteria from supurated cervical lymph node.

## Results

The smear result was positive for acid-fast bacilli and culture of specimen resulted positive for mycobacteria fully susceptible to rifampicin, isoniazid and ethambutol, but resistant to pyrazinamide. M. bovis BCG was identified by using a commercial identification kit (Genotype MTBC kit-Hain LifeScience). The VNTR-MIRU genotyping and spoligotyping of the isolate DNA was performed subsequently. The child was sent by her parents abroad and the treatment outcome is not known to us so far.

## Conclusion

To our knowledge this is the very first case of laboratory proven BCG infection following vaccination in children in Albania. Although BCG is considered to be a safe vaccine, it should be kept in mind that complications related to BCG do occur especially in children with cellular immunodeficiencies. Prevalence of BCG disease in Albania is not exactly known.

## Disclosure of interest

None declared.

